# Long-term history of vehicle collisions on the endangered Nēnē (*Branta sandvicensis*)

**DOI:** 10.1371/journal.pone.0210180

**Published:** 2019-02-20

**Authors:** Christopher A. Lepczyk, Jean E. Fantle-Lepczyk, Kathleen Misajon, Darcy Hu, David C. Duffy

**Affiliations:** 1 School of Forestry and Wildlife Sciences, Auburn University, Auburn, AL, United States of America; 2 Department of Zoology, University of Hawai‘i at Mānoa, Honolulu, HI, United States of America; 3 U.S. National Park Service, Hawai‘i Volcanoes National Park, Hawai‘i National Park, HI, United States of America; 4 U.S. National Park Service, Pacific West Regional Office, Hawai‘i National Park, HI, United States of America; 5 Pacific Cooperative Studies Unit, Department of Botany, University of Hawai‘i at Mānoa, Honolulu, HI, United States of America; Sichuan University, CHINA

## Abstract

Millions of birds in the United States die annually due to vehicle collisions on roads. Collisions may be of particular interest for species of conservation concern, such as the endangered Hawaiian goose (Nēnē), which is endemic to Hawai‘i. Using a nearly 40-year dataset of Nēnē road mortality in and around Hawai‘i Volcanoes National Park, we sought to answer the following research questions: 1) has Nēnē mortality changed over time? 2) are there times of the year in which mortality is greatest and does it relate to specific events in the species’ lifecycle? 3) does age at mortality differ over time, space, or sex? 4) given that existing mortalities appear to occur only in certain locations, do the number of mortality events differ across these locations; 5) does mortality rate show any density dependence? and, 6) are mortality rates related to numbers of visitors or vehicles? Between 1977 and 2014, a total of 92 Nēnē died from vehicle collisions; while absolute mortality increased over this time, the mortality rate remained the same. Similarly, average age of mortality increased over time, but did not differ by location or sex. Between 1995 and 2014, Nēnē population size and mortality rates were not correlated. Mortality was greatest in November and December (breeding season) and lowest in June. Most of the mortality occurred along just three stretches of road in and around the park, with the number of mortalities split about evenly inside and outside of the park. Furthermore, Nēnē mortality was unrelated to the number of visitors or traffic volume in the park. These findings suggest vehicle collisions are a growing concern for Nēnē, but that management actions to reduce mortality can be targeted at specific road segments and times of the year.

## Introduction

Anthropogenic sources of mortality represent a significant loss to many bird species across North America [1]. Direct sources of human-caused mortality range from window collisions to feral cats [2]. Vehicle collisions are a growing concern among causes of direct anthropogenic mortality [3–5]. Although bird-vehicle collisions resulting in injury or death of birds occur on roads [4], not all species of birds suffer negative population repercussions from roads [6].

Recent estimates indicate that between 89 and 340 million birds in the United States and [7] and ~9 to ~19 million birds in Canada [8] die each year due to vehicle collisions on roads. While vehicle-caused bird mortality differs by taxonomic group and season [8], information about mortality rates in terms of regional, seasonal, and taxonomic patterns is still somewhat rare [7]. However, several attributes have been correlated to the probability of vehicle mortality across species. For instance, bird species with a large brain relative to their body size tended to avoid vehicles more often than species that had a small brain [9]. Likewise, birds that forage, nest, or roost near roads have a greater likelihood of collision [3,10].

For species of conservation concern, vehicle collisions may be one more factor hindering recovery efforts. In fact, three endangered bird species in the US are considered to have population level impacts due to vehicle collisions: The Florida Scrub-Jay (*Aphelocoma coerulescens*), Audubon’s Crested Caracara (*Polyborus plancus audubonii*), and the Hawaiian Goose or Nēnē (*Branta sandvicensis*; [10]). Amongst these three species, one of particular concern is the Nēnē, which is endemic to the Hawaiian Islands and faces a host of other threats (e.g., introduced mammalian predators, habitat loss; [11]). Originally occurring across a number of the islands in the archipelago, the population was estimated at over 25,000 in the late 1700s but was reduced to less than 50 individuals by the 1940s [12]. During the 1950s, captive breeding was initiated to begin recovery of the goose. Currently the statewide population is estimated at over 3,000 individuals [13], but the species still faces many challenges to recovery. A survey of 300 opportunistically collected Nēnē carcasses in Hawai‘i found that vehicle collisions accounted for 5.7% of all deaths where the cause of mortality could be identified [14]. Notably, these mortality figures are unlikely to be representative of overall mortality rates because they were based on opportunistic sampling, and carcasses from vehicle mortalities are rarely sent in for necropsy as the source of mortality is known. In a recent assessment of resighting and carcass collection data at Hawai‘i Volcanoes National Park (HAVO), where approximately 90% of the park’s population is banded and resighting effort is high, vehicle collisions accounted for 14% of mortality from 2009–2016 (K. Misajon, unpublished data). Likewise, a previous assessment of mortality factors indicated that vehicle collisions were the leading cause of death [15]. Nēnē are amongst the most endangered waterfowl in the world [16] and are a long-lived species with relatively low recruitment rates at HAVO [15]. Thus, increased adult mortality may have serious implications for the population. Evaluating and understanding causes of mortality are critical for developing and improving management methods that can contribute to species recovery [14].

To aid our understanding of Nēnē mortality and help reduce vehicle strikes, we examined the trends and circumstances of vehicle collisions. Specifically, we sought to answer six basic questions. First, has Nēnē mortality due to vehicles changed over time and space over the past 40 years? Second, are there times of the year in which vehicle-caused mortality is greatest and does it relate to specific events in the species’ lifecycle? Third, does age at mortality differ over time, space, or sex? Fourth, given that existing mortalities appear to occur only in certain locations, do the number of mortality events differ across these locations? Fifth, does the vehicle-caused mortality rate show any density dependence? Sixth, are mortality rates related to numbers of visitors or vehicles?

## Methods

**Fig 1:**
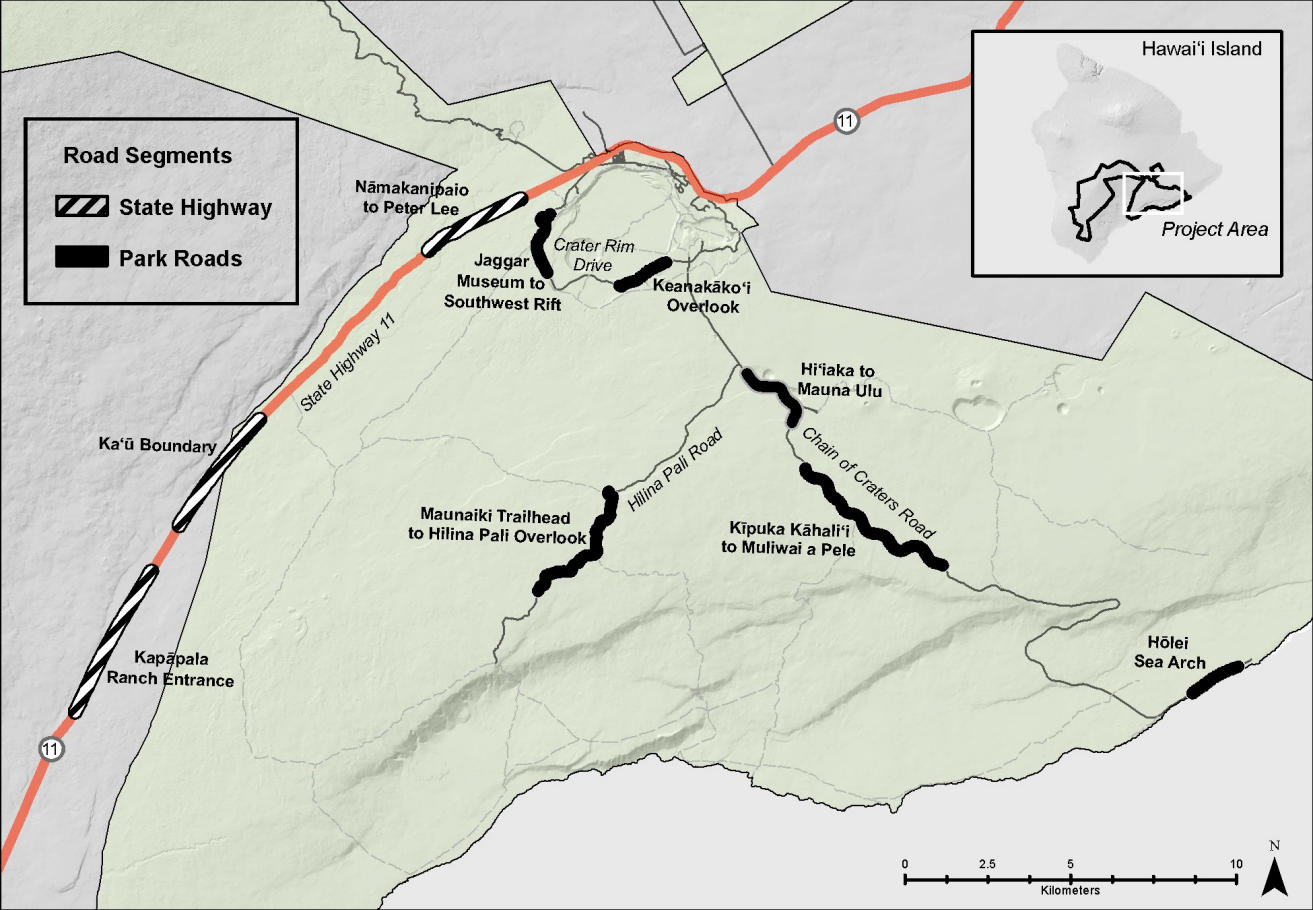
Hawai‘i Volcanoes National Park (HAVO) study region with major road segments.

The US National Park Service has kept records of Nēnē road mortality events from 1977 to 2014 in and adjacent to HAVO (Fig 1) on the island of Hawai‘i. Road mortalities were reported to HAVO both by the general public and park staff year round, as the species is non-migratory. These mortalities were then relayed to a biologist, who followed up to verify and document the event. While records of vehicle-related mortality likely are incomplete (e.g., they could miss birds that were hit and unreported), they likely represent most of the vehicle strike deaths within the study area for several reasons. Specifically, Nēnē are well-known in Hawai‘i (e.g., they are the State Bird), the park’s Nēnē restoration work is familiar to the community by virtue of periodic articles in the local media, the national park is a visible and well-known neighbor and easy point-of-contact for residents and staff commuting through the study area, park staff receive regular information about Nēnē management and are familiar with the park Nēnē biologist, and usually there is someone available to respond quickly to reports of dead geese. Thus, we believe the data are an unbiased sample of Nēnē killed by vehicles over the course of the study. The park also tallies numbers of visitors and, since 1990, numbers of vehicles entering through its two entry points.

From the long-term road mortality data in and around HAVO, we quantified the total number of Nēnē killed by vehicles between 1977 and 2014 by year, age, sex, location, and month. In the case of age, we classified goslings as 0.5 years old for analysis and used the minimum age of individuals in cases where a bird could be older. Both mortality and age data were log transformed to meet assumptions of normality for regression analysis. We evaluated if mortality and age at mortality had changed over the nearly 40 years using simple linear regression. Further, we evaluated if the number of individuals killed varied by age using simple linear regression. We evaluated if age at mortality differed by location inside or outside of the park as well as sex using a t-test. Similarly, we evaluated if age at mortality differed by specific road segment using ANOVA. To determine if mortality differed by location, we compared mortalities among the nine road segments, which ranged from 1.6 to 5.6 km in length, based on where the mortalities were documented, and evaluated if total mortalities over time were similar by location using a chi-square test. Likewise, we evaluated monthly totals to determine if road mortality rates differed using a chi-square test. For years in which both Nēnē population estimates and road mortality data were available (1995 through 2014) we calculated the percent of the population killed by vehicles each year. We evaluated if mortality and population size were related, as well as if the annual percent mortality changed over time, using simple linear regression. Likewise, we evaluated if annual Nēnē mortality was related to the annual numbers of vehicles or visitors using simple linear regression. We carried out all analyses in Systat 13, with a *P* < 0.05 considered significant.

## Results

**Fig 2:**
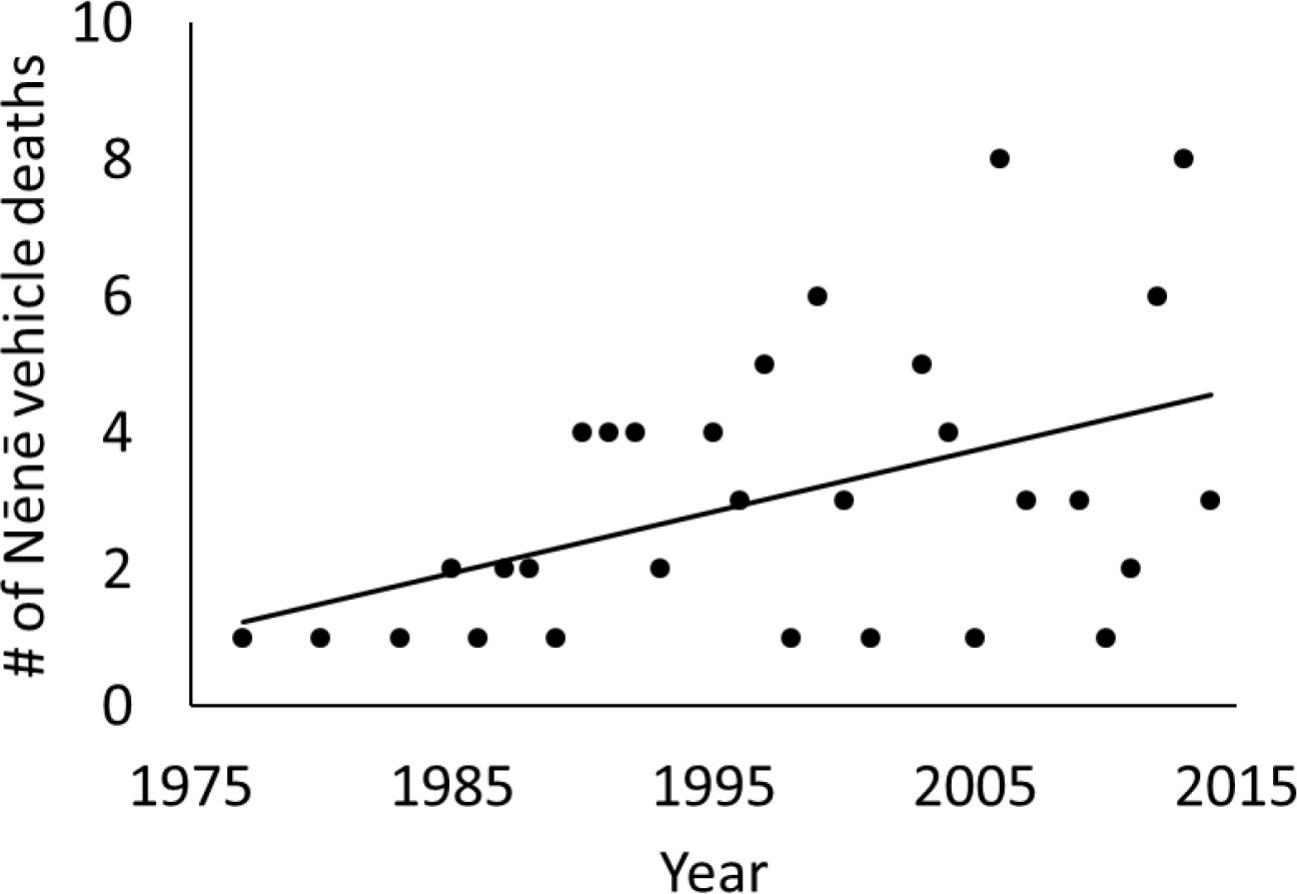
Annual Nēnē mortality due to vehicles from 1974–2014.

**Fig 3:**
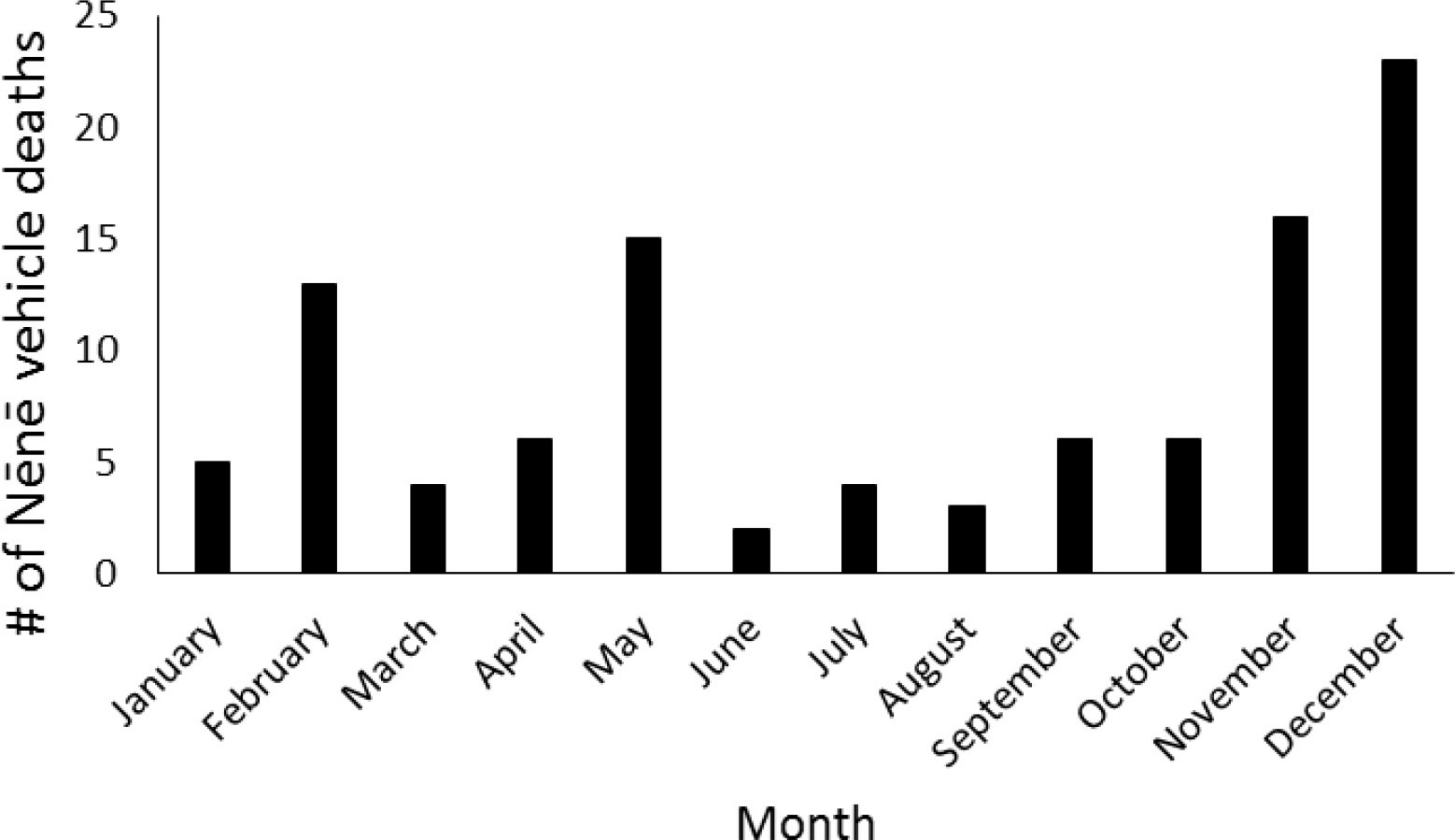
Monthly Nēnē mortality summed over the 40-year period.

**Fig 4:**
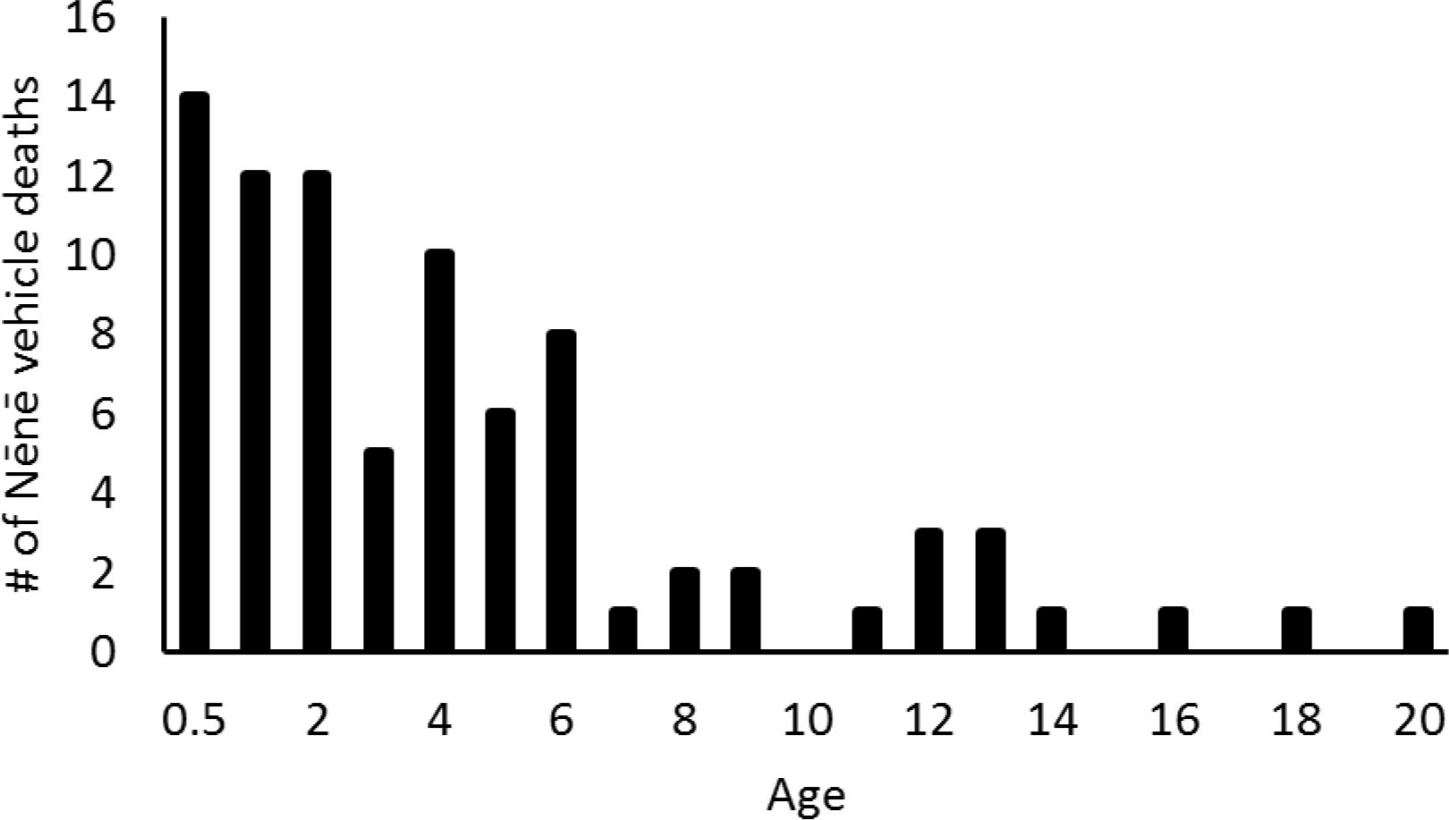
Age of Nēnē at time of mortality. Because not all goslings could be aged to exact month we considered them all 0.5 years old.

**Fig 5:**
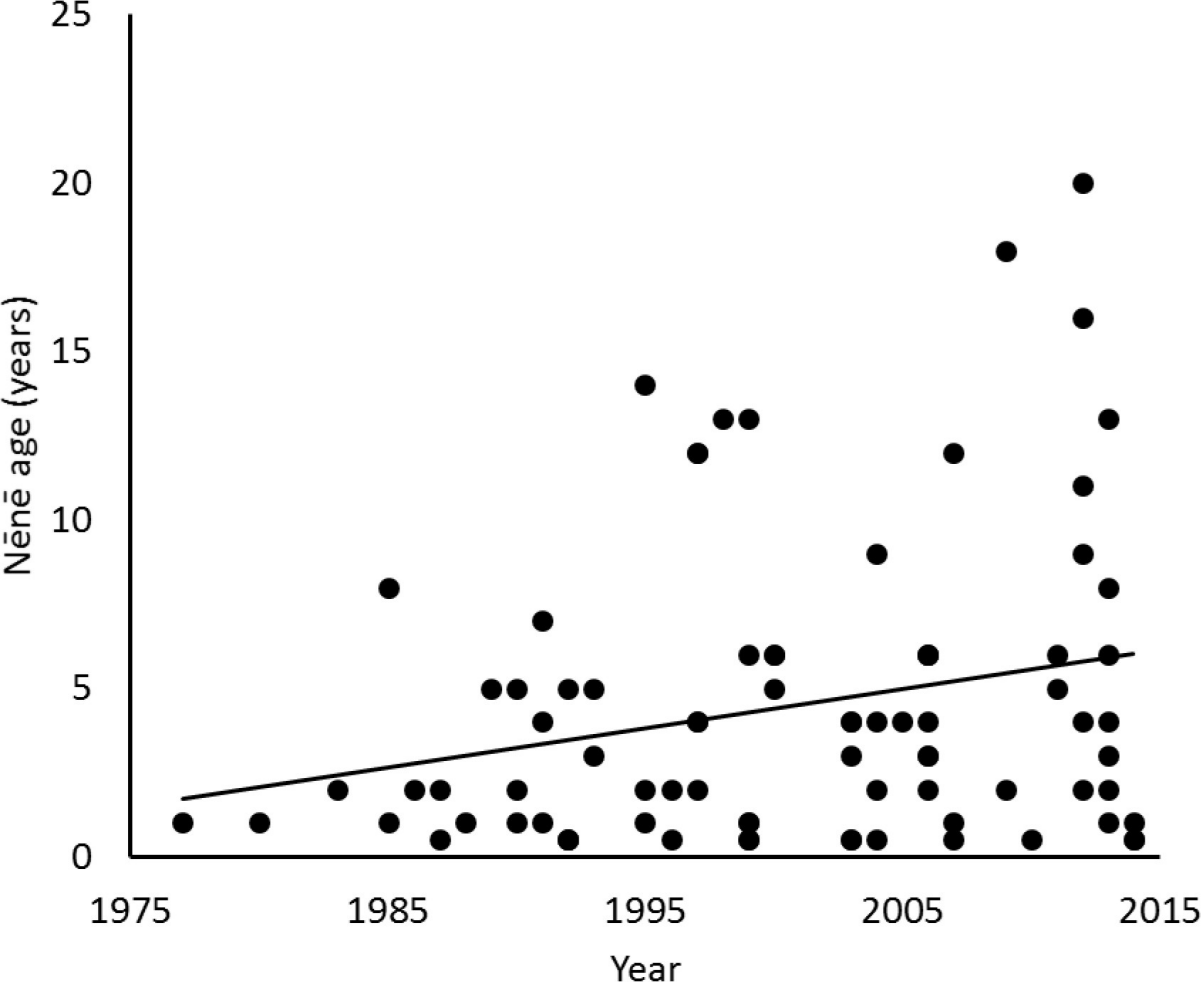
Age of Nēnē at time of mortality compared to year when mortality occurred.

**Fig 6:**
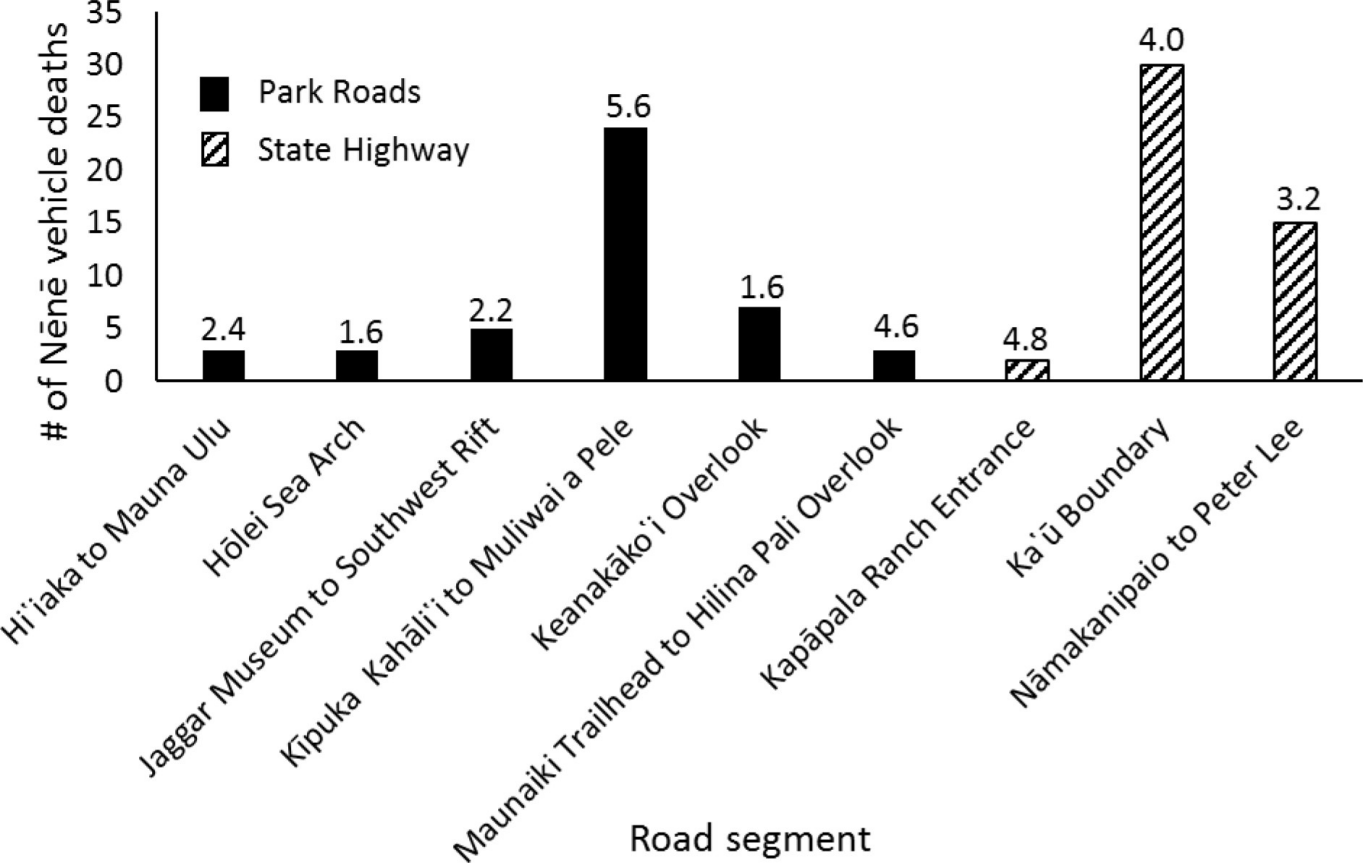
Total Nēnē mortality by road segment within the park and location of the segment inside or outside of the park. Road segment lengths (km) are denoted above each bar.

**Fig 7:**
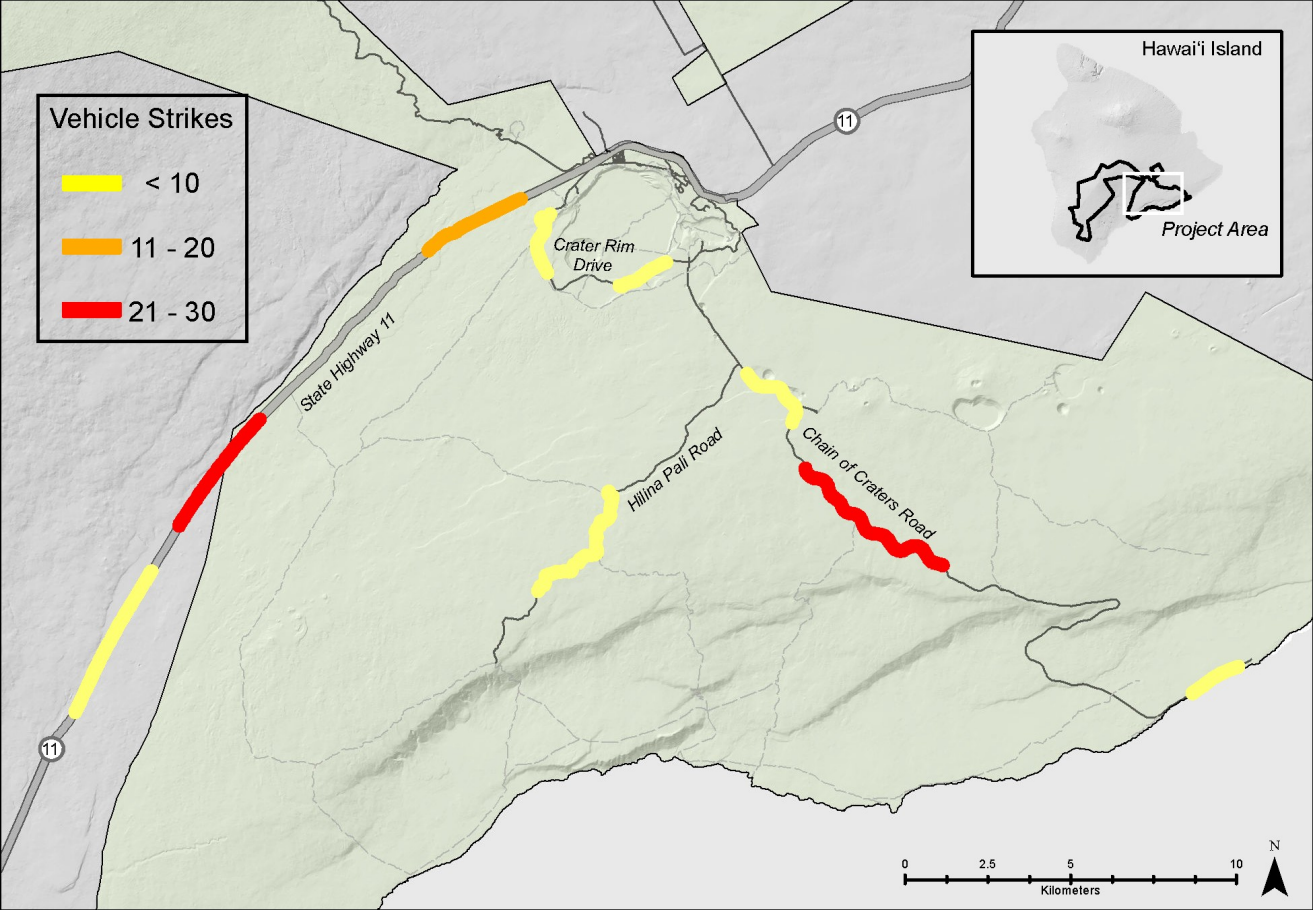
Locations of Nēnē vehicle strikes.

Between 1977 and 2014, 92 Nēnē were reported to have died from vehicle collisions in and adjacent to HAVO, for an average of 2.42 ± 2.21 (SD) birds per year. Over this nearly 40-year period, Nēnē vehicle collisions increased significantly (F_1,28_ = 6.96, p = 0.013, r^2^ = 0.20; Fig 2). However, the average annual percent mortality rate remained unchanged over the years in which population estimates were conducted (F_1,16_ = 0.08, p = 0.79). Mortality also varied significantly by time of year (χ^2^ = 40.26, df = 11, p < 0.001), with December and November being the months of greatest mortality and June being the lowest (Fig 3). The average age at mortality for Nēnē was 4.47 (range 0.5–20, n = 83) years old, with the number of mortalities decreasing significantly by age (F_1,15_ = 29.23, p < 0.001, r^2^ = 0.66; Fig 4). Males (n = 33) and females (n = 32) were killed in nearly equal numbers. The average age of Nēnē at the time of collision increased over time (F_1,81_ = 98.14, p = 0.025, r^2^ = 0.06; Fig 5). However, age at mortality was unrelated to whether the collision occurred inside or outside of the park (t = -0.16, df = 81, p = 0.88), the specific road segment (F_8,74_ = 12.09, p = 0.79), or the sex of the bird (t = 0.45, df = 63, p = 0.66). The number of mortality events was split nearly evenly between roads inside (n = 45) and outside (n = 47) the park. However, three of the nine road segments accounted for 75% of the mortality events (Fig 6), resulting in significantly different numbers of mortalities by road location (χ^2^ = 84.67, df = 8, p < 0.001; Fig 7).

**Fig 8:**
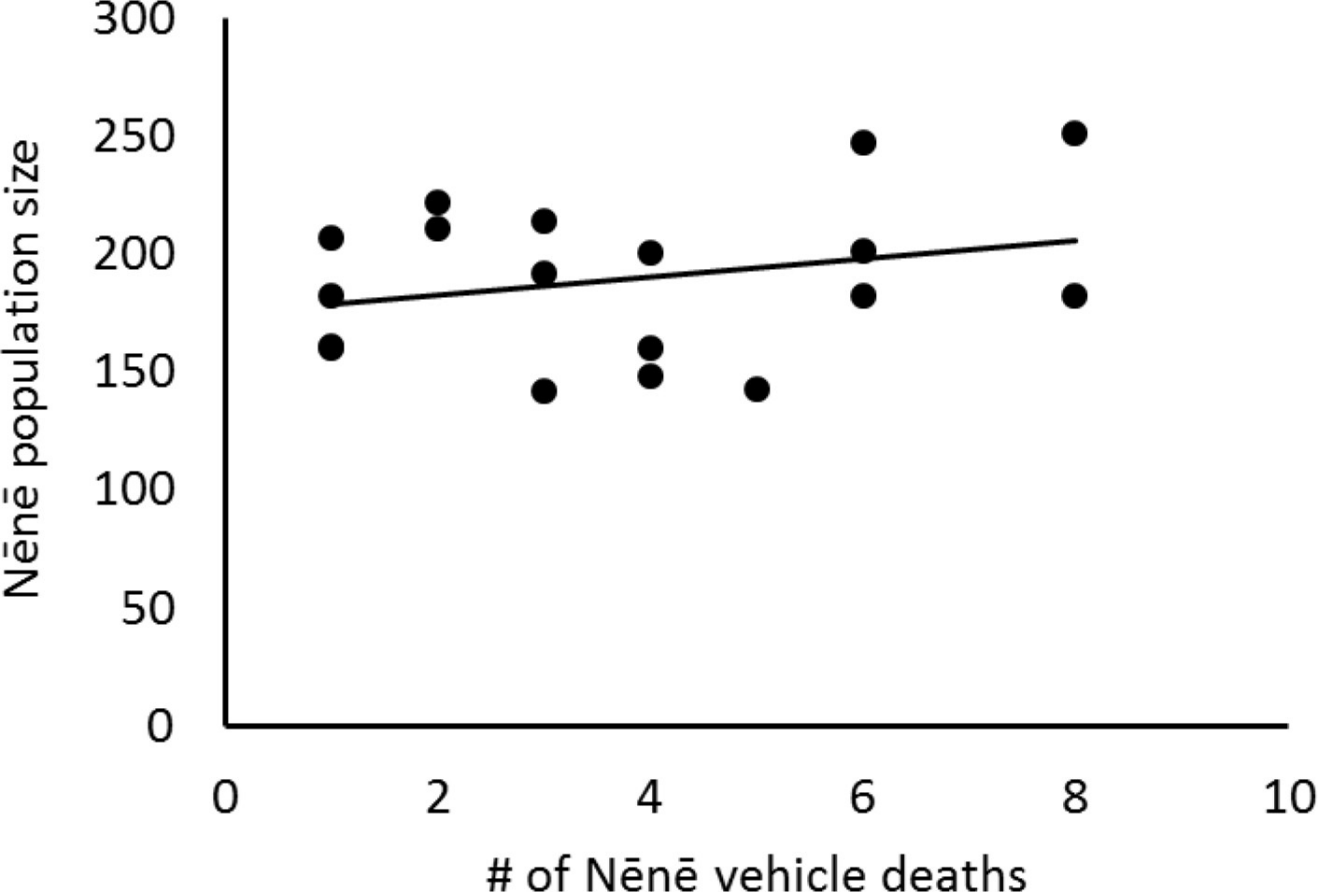
Nēnē population size versus vehicle-related mortality.

Between 1995 and 2014, the Nēnē population increased significantly from approximately 150 individuals to 265 individuals (F_1,16_ = 17.17, p = 0.001). However, population size and vehicle-related mortality rates were not correlated ((F_1,17_ = 0.68, p = 0.42; Fig 8). Although visitor numbers slightly increased over time since the late 1970s, the increase was not significant (F_1,28_ = 1.33, p = 0.26). Likewise, there was no relationship between the number of visitors in the park and the number of mortality events in a given year (F_1,28_ = 0.69, p = 0.41). On the other hand, since vehicle data collection began in 1990, the number of vehicles entering the park at Gates 1 and 2 increased 50% (from 451,531 vehicles/yr to 679,055 vehicles/yr), indicating a statistically significant rise in traffic (F_1,20_ = 14.15, p < 0.001). However, this rise in vehicle volume was not correlated to the number of Nēnē mortalities (F_1,20_ = 1.39, p = 0.25).

## Discussion

Among our six research questions, we found support for three of them. Specifically, Nēnē mortalities due to vehicle collisions increased over time. Second, Nēnē mortality varied within years, with the greatest numbers occurring in December and November, corresponding to their breeding season [11]. Third, Nēnē demonstrated spatial aggregation in mortality, with the majority of collisions occurring on just three road segments. On the other hand, we found no relationships between mortality and Nēnē population size, the number of park visitors, or the number of vehicles entering the park. Likewise, while the average age at mortality rose over time, there were no relationships with age and specific road segments, location inside or outside the park, or sex.

Though relatively few bird-vehicle collision studies have been conducted, our findings of spatiotemporal hotspots in mortality are consistent with similar studies (e.g., [17–18]). Specifically, most of the mortalities are occurring in the months during breeding and fledging and mainly along three road segments. While the number of Nēnē-vehicle mortalities increased over the nearly 40 years investigated, during the last 20 years the annual rate of mortality did not. That is, from 1995 onward, the percent of the population killed due to vehicles was similar. The lack of correlation of mortality with bird population size, traffic volume, and number of visitors suggests that there may not be a direct causal relationship with mortality. Alternatively, the lack of correlation could simply be an artifact of a small sample size or random chance events. Notably, while traffic volume has been related to mortality rates for some animal species [19], for birds, no such correlations have been found in other studies [17,20]. Regardless, the rise in vehicle-caused deaths is troubling, as they could serve as an additive source of mortality for a species already facing a host of other anthropogenically related challenges to recovery [21].

Our findings have several important management implications for Nēnē. Management actions can target the three road segments where most mortalities occurred (Kīpuka Kāhali῾i to Muliwai a Pele, Ka῾ū Boundary, Namakanipaio to Peter Lee; (Fig 4), and also focus on the months when the majority of collisions occurred (November to February, May). Lastly, considering that about half of the deaths occurred outside HAVO’s management jurisdiction, on a state highway, reducing collisions across the landscape requires state and federal agencies to work together in developing and implementing conservation and recovery actions.

Based on investigations of Nēnē killed on roads and intensive observations of live birds during the course of annual monitoring, researchers noted that geese mainly frequent roads either to forage alongside and nearby, or to cross roads separating nesting sites from brooding habitats, often with flightless goslings. In some locations, the trek between nest and brooding site occurs once, shortly after hatching. However, in other locations, families may commute regularly, or periodically, between daytime foraging areas on one side of a road and night roost sites on the other. Strategies to reduce vehicle strikes may differ in these two circumstances, but likely will include actions from each of two generalized approaches to reducing wildlife-vehicle collisions: those attempting to change animal behavior and those focusing on changing driver behavior [10].

Signage is the most common way to change driver awareness and behavior. Though relatively few studies have evaluated the effectiveness of warning signs [22], driver simulation studies have demonstrated they raise driver awareness [23], and that when signs were erected, they resulted in reduced collisions for deer in the year after installation [22]. Furthermore, the use of temporary warning signs that had reflective flags and solar-powered flashing amber lights resulted in a marked reduction in collisions and resulted in reduced traffic speeds [24]. Use of more dynamic imagery in warning signs also may improve driver awareness and response times [25].

Additional approaches to changing driver behavior could be evaluated in key locations. These include temporary digital signs that can display speed limit, verbal or iconographic warnings and/or other information, and temporary digital speed feedback signs. Devices such as rumble strips to slow traffic also could potentially alert Nēnē to oncoming vehicles. However, reflectors designed to alert deer to vehicles appeared to have a diminishing effect over time [26]. Because approaches to alert and/or slow drivers, including signage and mechanical methods, have not been researched extensively regarding their effectiveness in reducing wildlife mortality, such mitigation techniques should be carefully evaluated if employed for Nēnē.

Among techniques focused on changing animal behavior, wildlife fencing is the single most recommended approach to reduce vehicle deaths [10,27–28]. Another technique is vegetation management, such as eliminating lush grass or other forage species on road shoulders and edges, to reduce the attraction of herbivorous birds. Vegetation management should occur concurrently with wildlife fencing to reduce bird activity within the road corridor. This combined method is currently in use on Hawai‘i Island along a one mile section of the Saddle Road (State Route 200), and a subsequent reduction in vehicle collisions with Nēnē has been observed (Joaquin Mello, pers. comm.). A third recommended approach is the use of underpasses, in which Nēnē could traverse beneath the road bed [29]. Although fences and underpasses could be useful in areas where birds must cross on foot, such as along segments separating nesting from brooding sites, this technique may not be feasible in all areas. However, due to their widespread success and recommendation, small scale testing within the park could be an initial step to inform further mitigation actions for the species. In particular, before-after control impact (BACI) evaluation of the efficacy of any mitigation actions, preferably over multiple years [28], can help determine if further measures are needed.

As we have demonstrated, Nēnē mortality from vehicles has been increasing over time, but has been occurring at times and locations that management can target in order to reduce mortality. Studies such as ours may be of particular value in using patterns in vehicle mortality to inform management actions in the context of the species’ underlying biology and ecology. Finally, our analysis of exceptionally long-term data on Nēnē road deaths helps address the dearth of research on vehicle collisions with both non-game species and smaller animals.

## Supporting information

S1 Table Lepczyk et al. Nene vehicle collision data. (XLSX)
